# Investigating the psychosocial impact of COVID-19 on coastal communities in East Sussex, UK: a qualitative analysis

**DOI:** 10.1136/bmjopen-2025-102031

**Published:** 2025-10-23

**Authors:** Nigel Sherriff, Alexandra Sawyer, Laetitia Zeeman, Lester Coleman, Sarah Kennedy, Jane Thomas, Joanne Bernhaut, Teresa Salami-Oru, Darrell Gale

**Affiliations:** 1 School of Education, Sport, & Health Sciences, University of Brighton, Brighton, UK; 2 Centre for Transforming Sexuality & Gender, University of Brighton, Brighton, UK; 3 Coleman Research and Evaluation Services, Hurstpierpoint, UK; 4 Public Health Team, East Sussex County Council, Lewes, England, UK

**Keywords:** COVID-19, MENTAL HEALTH, Public health

## Abstract

**Objective:**

COVID-19 led to significant economic and psychosocial impacts on individuals and their local communities. This research aimed to investigate the psychosocial impacts of the COVID-19 pandemic on a diverse range of individuals living in coastal areas in East Sussex, UK, including adverse, unexpected and positive outcomes.

**Design/Setting:**

This cross-sectional qualitative study used semistructured interviews conducted remotely between December 2020 and March 2021, referred to as the third lockdown. Interviews were recorded and transcribed. Thematic analysis was used to identify, describe, and analyse themes and patterns within the data.

**Participants:**

Purposive sampling was used to recruit 25 participants living in East Sussex, to include a range of ages (above 18 years), genders, race/ethnicities, identities (eg, lesbian, gay, bisexual, trans or intersex) and social backgrounds.

**Results:**

The pandemic was described as a significant life-changing event, with people saying their plans had changed ‘overnight’ and that their life was ‘on hold’ or it had lost its ‘infrastructure’ during the pandemic. Immediate changes to social lives, education, future plans, work, and housing were evident. These changes were felt particularly by those shielding (due to underlying health conditions) and people placed in emergency accommodation. Significant areas of impact were around family, friends, leisure, mental health, health-related behaviours, and employment. Some participants reflected on positive impacts around an increased sense of well-being or the restrictions affording time and flexibility to re-engage with their families. Adverse impacts around reduced friendship groups and the disruption to family life were often mitigated by developing strategies that helped adapt to new situations.

**Conclusions:**

Residents of coastal areas in East Sussex were impacted widely and differentially. Both positive and challenging impacts related to the COVID-19 pandemic were experienced unequally, potentially exacerbating health inequalities in coastal communities who were already at risk. Long-term strategies should consider the vulnerabilities of people living in coastal areas in planning for future health crises.

**STRENGTHS AND LIMITATIONS OF THIS STUDY**

- This study explored the psychosocial impacts of the COVID-19 pandemic on individuals living in coastal areas using a cross-sectional qualitative study design.
- A purposive recruitment approach meant it was possible to recruit a diverse sample of adults from coastal communities.
- The sample size for this study was small, meaning that it is beyond the scope of the research to offer broader recommendations for public health.
- Due to the study taking place during a pandemic, opportunities for patient and public involvement were limited.

## Introduction

Novel COVID-19 was first reported in December 2019 and spread rapidly, being characterised as a global pandemic by the WHO in March 2020. The first case of COVID-19 was confirmed in the UK on 31 January 2020, and up until March 2023, it was reported that over 500 million COVID-19 tests had been taken and over 9 in 10 people had at least two vaccinations.^1^ Overall, the UK recorded 220 721 deaths mentioning COVID-19 on the death certificate.^2^ Initially, policy responses by the UK government relied mainly on non-pharmaceutical interventions and successive national lockdowns from 26 March 2020 to 8 March 2021 when England began a phased exit from the final lockdown. Lockdown measures included enforced social distancing (eg, working from home, prohibiting public gatherings, closing schools) and social isolation (eg, remaining indoors).

COVID-19 and the subsequent government restrictions led to dramatic economic, social and personal changes with significant psychosocial impacts on individuals. The UK Household Longitudinal Study, examining adult mental health problems, indicated that the prevalence of mental health problems was significantly higher post lockdown compared with prelockdown.^3^ A review showed that fears of infection, fears about infecting others, future uncertainty, boredom, frustration, social isolation and hopelessness were the most profound psychological impacts of the pandemic.^4^ Another study (using an online survey of n=1,963) reported that 27% of participants reported loneliness during the pandemic.^5^ Risk factors for loneliness were being younger, separated or divorced, meeting clinical criteria for depression, difficulties emotionally regulating and poor-quality sleep due to COVID-19. Other studies have also shown that some groups may be more vulnerable than others to the psychosocial effects of pandemics, including those at heightened risk for COVID-19 infection (eg, older people, people with compromised immune systems), socioeconomically disadvantaged groups and those with existing medical conditions.^6 7^

Coastal towns are considered vulnerable due to the compounding of risk factors and the wider determinants of health including, for example, a high proportion of people over 65 years, poor quality housing and high levels of deprivation.^8^ Furthermore, many seaside areas report poor outcomes around measures of productivity, growth and employment opportunities.^9^ The economic impact of COVID-19 on coastal areas was severe, placing additional pressures on communities who rely heavily on employment opportunities and income from tourism and hospitality.^10^ In East Sussex, the three largest towns are seaside towns,^11^ two of these (Hastings and Eastbourne) also face some of the highest levels of deprivation in the county.^12^ There were concerns around the COVID-19 pandemic exacerbating inequalities between coastal and non-coastal areas even wider,^10^ ^12^ as was reported with vulnerable groups across the UK including people with a disability, ethnic minority communities, and care home residents.^13^,^15^

Qualitative research carried out on adults during the COVID-19 pandemic on UK coastal communities as reported in this paper can complement epidemiological data by providing insight into people’s lived experiences of pandemics, social responses and unexpected consequences.^16^ For instance, large-scale qualitative studies across countries and ages have highlighted the psychosocial impact of COVID-19 on adults’ mental health.^17^ Interviews with adults in the UK report contrasting experiences depending on people’s intersecting socioeconomic circumstances.^18^ Older adults (>70 years) reported threats to mental health and well-being; however, some participants manage this by adopting coping behaviours or activities.^19^ Adults with existing mental health conditions commonly report how these were further exacerbated by the COVID-19 pandemic, although some participants found they were able to adapt and form new routines,^20^ ^21^ highlighting the need for an indepth understanding of how to provide effective and tailored support for vulnerable population subgroups.

The COVID-19 pandemic has highlighted a disproportionate impact on vulnerable populations, and identifying gaps to understand this has been identified as a public health research priority.^22^ However, there is limited qualitative research on adults living in economically deprived UK coastal communities. The aim of this study therefore, was to explore the psychosocial impacts of the COVID-19 pandemic on adults living in coastal communities in East Sussex, in the South-East of England, at a time when the county was entering into a third lockdown. A better understanding of how the COVID-19 pandemic affected communities living in coastal areas can inform future pandemic preparedness tailored to these areas.

## Methods

The research design was a cross-sectional qualitative study using semistructured interviews (see online supplemental file 1 for topic guide).

### Setting

East Sussex has a population of approximately 550 700 with a significantly higher proportion of people aged 65+ years than both England and the South-East.^12^ The county comprises the Boroughs of Eastbourne and Hastings and the Districts of Lewes, Rother and Wealden. East Sussex is predominantly rural; however, 55% of the population live in coastal areas.^12^

East Sussex has 22 neighbourhoods out of 329 (6.7%) in the most deprived decile. Two lower super output areas (LSOAs) are among the most deprived 1% in the country (both of which are in Hastings) and another eight are among the most deprived 5% of LSOAs, all of which are also in Hastings except one LSOA, which is Bexhill in Rother.^23^

### Recruitment and sample

Diverse participants (n=25) were recruited via purposive sampling including a range of ages (above 18 years), genders, race/ethnicities, identities (eg, LGBTI), and social backgrounds who were living in East Sussex at the time (online supplemental file 2). Selection of participants was informed by local authority insight concerning those most likely to be impacted negatively by the restrictions and was based on a balance of the following criteria: furloughed worker or unemployed due to COVID-19, shielding during lockdown, LGBTI, older teenager or young adult in education, single parent household; living in temporary accommodation, rough sleeper now in temporary accommodation, working from home and women who have given birth during the pandemic. Participants were recruited during two phases: phase 1—via professionals and contacts known to the research team (n=10) and phase 2—under-represented groups were targeted via purposive snowball sampling (at the end of interview participants were asked if they knew of anyone who would be interested in participating), via social media (eg, Facebook and X); and via voluntary sector organisations (n=15) (eg, Rough Sleeper Initiative). Participants had to be able to give informed consent and to understand and speak English to take part in the study (interviews were in English due to time and resource limitations).

A semistructured topic guide was used to generate the qualitative data allowing participants to have flexibility in their answers and identify or explore further areas as required. It was developed collaboratively by the research team in partnership with the public health team at East Sussex County Council. This was particularly important to ensure that individual, community and wider public health impacts were captured, reflecting the social and wider social determinants of health (eg, education, employment, housing). The topic guide was reviewed and refined iteratively after each interview. Designing the schedule in this way meant it was intentionally broad to capture a wide range of impacts of the COVID-19 pandemic across diverse participants, but also that it could be used as an ‘aide memoir’ creating a sense of informality in the discussions and allowing people to share their experiences including those that were not anticipated in advance.

## Data analysis

With participants’ permission, all interviews were digitally audio recorded, quality checked, transcribed verbatim and then anonymised including participant names being replaced with pseudonyms. Qualitative thematic analysis was applied to the data which allowed themes to be identified both inductively (unanticipated findings arising from the interviews) and deductively (based on the project objectives). The approach to thematic analysis was based broadly on Braun and Clarke^24^ to identify, describe and analyse common themes and patterns in the data. The analysis focused on the generation of themed summary sheets for each participant undertaken by two researchers, with a third researcher identifying and deriving emerging themes and explanations across all datasets. Data collection continued until thematic saturation was reached, meaning no new themes were being identified and existing themes were well-developed and supported across the dataset. We did not use assess coding reliability or intercoder consensus, as these approaches are arguably more aligned with positivist paradigms. However, to enhance analytical rigour the team met regularly to discuss themes and resolve any discrepancies. Findings are illustrated using quotes from the participant interviews.

Remote interview options were offered (eg, landline telephone, mobile, Microsoft Teams), depending on participant’s preference and technology available. If using video conferencing software, participants were given the option of joining with or without visual functionality. Due to institutional data collection guidelines, in-person data collection could not be conducted, and interviews had to be conducted remotely. Participants were therefore asked to complete a consent form either via email or to provide verbal consent which was then audio-recorded (a routine procedure for telephone/online interviews where all points on the consent form are read out and individually agreed to by the participant). Written information about the study was also provided as part of the consent process in a participant information sheet which included the following statement ‘…*results may also be presented at conferences and published in academic, peer-reviewed journals. Reports may include direct quotes from interviews. However, any names or other identifying information will be removed. A summary of the results can be sent to you if you wish to see them. You will not be personally identified in any reports or publications of the research*.’

21 interviews were conducted over the telephone and four using Microsoft Teams. All interviews were conducted in a quiet room where participants could not be overheard and where there was no risk of interruption. Interviews lasted approximately 1 hour. All participants were offered a £10 voucher as a thank you for their time.

### Patient and public involvement

As this study took place during the COVID-19 pandemic, including during the associated periods of lockdown in the UK, it was not feasible to conduct patient and public involvement (PPI) activities. Therefore, we confirm that patients/participants were not involved in the study design, conduct, reporting or dissemination plans of this research. In partial mitigation, the research team met regularly with the project commissioners and the wider local authority public health team.

## Results

25 participants participated in the study (age range: 18–71 years, 60% males, 84% white British or other. See online supplemental file 2 for demographic details. The results are presented according to the five key themes (first recollections of COVID-19; reducing the risk of transmission; concerns about transmission to others; initial impacts of COVID-19; specific impacts of COVID-19) and five subthemes (family, friends and leisure; health-related behaviour; mental health; health and social care appointments and employment).

### First recollections of COVID-19

Participants recalled the time when the first cases of COVID-19 arrived in the UK with many people thinking that the disease was unlikely to be serious. People frequently compared it to previous virus outbreaks like severe acute respiratory syndrome or Bird Flu (H5N1 virus) and could never foresee the impacts that had arisen, despite the original government estimates:

I thought it was just going to blow over. It wasn’t going to upset me, it wasn’t going to come close to me, sort of quite naïve. (Amelia, 22 years, finished college and furloughed).

The notable exceptions to the above were from a GP who anticipated the potential severity of the virus as it first developed:

At the very start of it… I probably was more aware of personal infection risk in places like supermarkets and public buildings and touching what you need to touch and not touching what you don’t need to touch. I probably became more aware of it as talk of it began to circulate. And obviously I was fairly professionally connected to this, so I knew which way it could go and was over aware perhaps quite early on about the potential for what could happen. (Phil, 55 years, employed).

### Reducing the risk of transmission

All participants reported that they had taken steps to reduce their risk, usually around the time of the first lockdown. Most said they had been following the rules of sanitising, keeping distance and wearing masks. This also included passing safety messages to their children:

There’s been instances when you're in a supermarket and places where it’s virtually impossible to maintain the distance. But we make sure we’ve got our masks and we're very good in this household, we're making sure we wash our hands as soon as we get in the house. And we've got hand sanitiser in the bucket load. (Kath, 32 years, left job voluntarily).

The extent of this risk reduction varied greatly from hand-washing and mandatory mask wearing to washing packaged food produce and regular hand sanitisation (especially when returning home) and generally staying housebound:

For example, washing my shopping, things that I wouldn’t have done before, making sure I have a mask, I've even got an FP2 mask because I know these things from my work. (Sue, 54 years, working at home).

### Concerns about transmission to others

Several people spoke about the concerns they had about being infected with COVID-19. This fear tended to express itself through risk-reduction behaviour such as rarely leaving the house and wearing a full face-shield protection. When venturing outdoors, some participants felt anxious about the risk of infection and potentially passing on the virus to others:

When we went back in July [school], I was really worried and nervous about it, I was getting quite anxious, and panicking really, I don’t want to get it, I don’t want my children and my partner to get it*.* (Darren, 30 years, furloughed).

### Initial impacts of COVID-19

The overriding response was that COVID-19 was a life-changing event, with people saying their plans had changed (sometimes ‘overnight’) and that their life was ‘on hold’ or had lost its ‘infrastructure’:

I’ve kind of gone into shut down and I sort of forget that I can do some things that are actually allowed. It’s all to do with the fact that my whole life infrastructure wasn’t there anymore. I didn’t have my job, I didn’t have the friends, my partner, who lives abroad, and that’s not proving very easy. (Sharon, 58 years, unemployed).

For the four people who were shielding, their responses tended to focus on their specific health conditions or restrictions. For them, the effects of the pandemic were immediate, often being confined to their home for long periods of time or rarely leaving the house for fear of bringing the virus home. In comparison, one participant who was also shielding was determined to maintain as normal a life as possible by walking with her family and close friends:

During lockdown I didn’t see anybody. When we were free to see people, it was just very occasionally and in the garden. Apart from my hospital appointments and stuff, I’ve only been out five times. (Meghan, 57 years, shielding).

Immediate changes to social lives, education, future plans, work and housing were evident. These changes were particularly felt by those shielding, and those who had been placed in emergency accommodation during the pandemic.

… funnily enough no [impacts]. Because there were three of us, we all had a bench each and we were quite away, we weren’t in town or anything, we were way out of the way, we were right next to the toilets, so we would keep hygienic and that…the Police found me on the beach, they did a sweep up, so they found me, they found me a hotel, which I think has happened to quite a lot of people. (Des, 40 years, former rough sleeper now in temporary accommodation).

### Specific impacts of COVID-19

#### Family, friends, and leisure

Many participants spoke of the positive aspects of the restrictions with the time and flexibility to re-engage with their families. Negative impacts on the immediate family included feelings of claustrophobia and children needing to be entertained:

The big impact I suppose has been on family, with four of us stuck in the house, not being able to get out very much… it can be really hard, we’re [family] getting on each other’s nerves basically, a lot, and there’s no release for it, there’s no outlet. (Richard, 63 years, working at home).

Family disruptions, compounded by children being off school and parent(s) working at home, were mostly temporary, with people adapting either their workspaces or taking steps to respect the needs of others. The impact on people’s friendship groups was a prominent feature of the conversations. For many people, friendship groups had reduced:

Very, very little, [contact with friends] and the reason being is that I do have friends, but the friends I have, they were all still working, so they were either keyworkers or people working from home, so they were juggling home schooling their kids, still working full time, so they actually had double the workload than they did before. Because they had to educate their children, and they had families and all this and I felt like they didn’t really have the time for me … it just felt I wasn’t part of anyone’s bubble. (Gemma, 41 years, furloughed).

Most friends seen in person were on a one-to-one basis, although wider groups connected through a range of social media apps. More positively, some people reported becoming closer to friends or reconnecting with friends who they had not seen for some time. Several people had increased their level of physical activity, mostly in the first lockdown, benefitting from the warmer spring weather:

Me and my mum went on walks a lot more. We went on really long hikes together and that was really nice. We got the chance to actually talk and looking back, if COVID hadn’t happened … there is a positive to it, that I got to appreciate more time just with my family at home. (Josie, 18 years, finished A-levels during pandemic, employed).

Several people spoke of discontinuing social activities such as choirs, book clubs, going to the pub and other activities. For many, these were activities where people would usually connect with others.

Choir was twice a week, because I was part of two choirs, now obviously I wasn’t part of it when I was abroad, but when I came back, I would try and do it. I’m part of a book group, and that was nice, we managed to meet once, so there was just five of us in the group, so it was alright, but now that’s on Zoom, and to be honest, it’s a bit miserable, I don’t like Zoom book club. (Sharon, 58 years, unemployed).

#### Health-related behaviour

Several people reported having more time and flexibility due to working at home or being furloughed. Positive shifts to health-related behaviours during the pandemic were reported (such as new fitness regimes or home cooking):

Cycling along the roads during the first start of lockdown was just magical. Cycling through the East Sussex lanes with no traffic at all was terrific. (Phil, 55 years, employed).

Some people reported that they drank more alcohol than usual on occasion or increased food consumption or were smoking more:

We have all drunk too much and eaten rubbish food to be honest. I think especially when we were in the first period when the weather was so nice, you know it was easy just to pour a drink, it was almost like we were on holiday at the beginning. And then the next couple of weeks it was like, you know I would say the alcohol intake has definitely increased. (Anna, 51 years, lost one of two part-time jobs and working from home).

#### Mental health

Most people’s mental health had been impacted by COVID-19 ranging from feeling *‘cross’*, *‘frustrated’*, *‘unmotivated’,* through to increased anxiety or feeling down:

Work, family, social life, kind of just all sort of runs into one, and I wouldn't say I felt depressed, just I definitely felt down, and it was sort of taking its toll on me … it’s been tense at times, and I think that it did take its toll on me. (Mick, 38 years, working at home).

Certain events contributed to worse mental health, such as entering a period of lockdown or knowing people or relatives who had been infected. People shared several coping and support mechanisms to improve or stabilise their mental health. These included: exercising (which was the most popular way to cope), writing down problems or worries, investing in new online courses, dog walking and getting comfort from pets, intense research into nutrition and embarking on a new diet, developing a daily routine of online calls, daily phone calls to a parent and trying to leave the house at least once a day.

#### Health and social care appointments

During successive lockdowns in the UK, health and social care appointments moved online. Most people viewed remote appointments as a suitable, more convenient alternative to face-to-face appointments, although this was dependent on the nature of the condition—whether it was routine or potentially more serious. Phone appointments were preferred over video, although video was perceived as useful in some circumstances (assuming the technology worked effectively), suggesting a choice of media may be beneficial to patients:

Definitely a phone call [for remote appointments]. I don’t like speaking on the video personally. Because my anxiety starts like kicking in, and they can see my facial expression. (Lauren, 18 years, lost her job).

#### Employment

The pandemic impacted participants’ employment in several ways, including reductions in income (particularly for the self-employed), changes in job role and increased work pressures or increased demands on their time due to working from home while home-schooling children, and enjoying the possibility of having some free time:

I’m looking forward to being alone [chuckling]. For some people they’ve had too much being alone, they can’t go out and meet other people. I’m looking forward to everybody else going out to school and work and having some time on my own. (Richard, 63 years, working at home).

Some people had become unemployed as a direct result of the pandemic. For all those who were furloughed, the experience was considered as enjoyable. People were grateful for the opportunity to be paid by the government while enjoying time at home, often using the time to pursue new activities or interests.

I was loving furlough; I was loving all the time off. I kept myself busy. I rarely got bored because I thought, ‘This is such an opportunity to have all this time off and to still be paid for it.’ I felt like I’d won the lottery when I needed it. And to this day I’m still very grateful for that time. (Amelia, 22 years, finished college and furloughed).

## Discussion

The COVID-19 pandemic with the related infection and mortality rates unfolded against a backdrop of varying social and wider determinants of health. It has been widely reported that COVID-19 exacerbated health inequalities across the UK^14 15^ and in coastal towns that were already at risk.^10 25^ Findings show that residents of East Sussex were impacted differentially and at different time points since the first lockdown in March 2020. The wide-ranging impacts of COVID-19 meant that various social determinants were altered for participants such as employment or income, social relations with family and friends, access to health and social care, and health-promoting activities.

The unprecedented measures taken by the UK government to contain COVID-19 meant that some businesses closed or scaled back their operations during lockdowns, risking increased levels of unemployment. Coastal areas reported some of the highest unemployment rates,^26^ and long-term sickness rates were highest among those with recent employment in retail, transport and hospitality sectors, which represent some of the biggest employers in coastal areas.^25^

During the pandemic, UK-based research found that employment, but more specifically income security, was associated with positive outcomes such as: improved use of recreational spaces, increased neighbourhood identification (where people felt part of their local community), better levels of exercise, and strengthening social networks.^27^ Conversely, people on lower incomes reported more tentative social networks, lower neighbourhood identification, and less engagement in exercise.^28^ While changes in employment and income were experienced differently by participants, some had increased their physical activity levels, motivated by good weather or used the time for education through online courses or research. For some participants, after successive lockdowns, new routines became essential coping strategies to stabilise mental health in the absence of their usual activities and social connections. Thus, considering measures to promote income security may aid facilitation of neighbourhood identity, commonality, and a shared sense of belonging.

Participants in the current study reported changes in income, employment and social networks during the COVID-19 pandemic, which impacted negatively on mental health, increasing feelings of frustration, anxiety and feeling down. Reports from longitudinal research support an overall increase in anxiety and depression in adults living in the UK during the pandemic.^3^ Subjectively perceived loneliness, experienced in the context of social distancing measures, was an important cause of both anxiety and depression.^29^ Both loneliness and impaired social networks played a substantial part in participants’ discussions around the impact of the pandemic on mental health. This echoes findings from a report including measures of loneliness in East Sussex which found that people who were living in the coastal towns of Hastings and Eastbourne were more likely to report feeling lonely some of the time or all of the time.^12^ This was more prevalent in people who: spent more time at home because of health or mobility issues, were out of work, were living with a disability, single person households and single parents. Participants in the current study who were shielding reported being confined to the house and not seeing people for long periods of time, which placed additional pressures on their mental health. Therefore, how the intersections of identity (eg, shielding and elderly) create additional layers of vulnerability is an important consideration for future qualitative research.

Research indicates that COVID-19 may compound pre-existing psychosocial problems in the general population.^28^ The COVID-19 Mental Health and Wellbeing Surveillance Report (2022) detailed the changes in levels of psychological distress in line with the enforcements and lifting of lockdown restrictions.^30^ Longitudinal studies reported that worsening of mental health did not improve with the lifting of lockdowns^31^ and measures of well-being have not returned to pre-pandemic levels, driven by reporting of higher anxiety levels in women.^32^ This was evident from the data in the current study where participants experienced both adverse and beneficial impacts of the pandemic, with some developing strategies over time to mitigate or amplify these impacts. Exercising was the most frequently used coping strategy; however, journalling, education, interacting with pets and daily calls to friends or family were also beneficial. Adapting to the highs and lows of the pandemic was reported across studies in young black and mixed ethnic groups (12–17 years),^33^ older adults (>70 years)^19^ and in people with a mental health condition.^20 21^ Another study reported that parents managed compounding stressors by recognising the need for self-care to build resilience, which included spending time in nature and together as a family.^34^ While studies report some positive outcomes from the pandemic, some data were collected during the earlier stages of the pandemic, rather than retrospectively. Future research should explore the long-term support needed for people managing mental health conditions and address existing mental health inequalities in the recovery from pandemic in preparation for current and future economic challenges.

Further literature shows that lockdown appeared to widen some kinds of inequality (eg, socially disadvantaged groups) but not all.^35^ These findings are consistent with historic experiences of pandemics where the most disadvantaged communities experienced higher rates of infection and mortality.^14^ The follow-up report from the COVID-19 impact enquiry reported higher mortality and lower vaccination rates in poorer areas.^32^ People are also facing additional challenges: the stress of the cost-of-living crisis, long-term health conditions keeping many people out of work, and education gaps due to lost learning which are disproportionately impacting poorer families.^32^ Furthermore, long COVID-19 was more prevalent in older people with pre-existing health conditions who were living in deprived areas.^32^ Providing generic messaging to shield was reported to be unhelpful when people were reporting worsening levels of stress and anxiety,^36^ and people who were shielding experienced poorer mental health and life satisfaction.^37^ The current study findings reflect these challenges; however, it is important to consider how living in economically deprived coastal areas could amplify these challenges through geographical isolation confounded by poor transport links, high rates of unemployment and the long-term impacts of poor mental health.

### Strengths and limitations

A key strength of this study was the inclusion of a diverse sample of adults from coastal communities at a time when people could reflect over the whole pandemic and the lockdown measures that were implemented. Additionally, this is one of the few qualitative studies that explore the psychosocial impact of the COVID-19 pandemic on people living in coastal areas, with data captured during the third lockdown. While this diversity provides rich insights into the impacts of COVID-19, it limits the ability to draw comparisons within and across specific subgroups or to explore fully how intersecting identities might impact on people’s experiences. Furthermore, other prominent members of coastal communities (eg, older people in care homes and other socially vulnerable groups such as refugees/asylum seekers and people from Gypsy, Roma and Traveller communities) were not included in the study. These groups would have had different experiences of the pandemic and therefore, as the long-term psychosocial impacts continue to unfold, it is important to prioritise capturing the long-term impacts of COVID-19 across additional vulnerable communities.

Interviews were conducted online, which can reduce the effectiveness of non-verbal cues. However, the interviewers used vocables such as ‘hmm-huh’ to demonstrate their attentiveness, which helped the discussion to flow. Online and remote methods of data collection can also help minimise power differentials between researchers and participants and may encourage participation from individuals or groups who are less likely to participate in face-to-face research, due to accessibility and anonymity. To support rapport building, researchers offered participants the opportunity to speak informally prior to the interview. During remote interviews, active listening and clear communication were used. Participants were informed they could pause or withdraw at any time and were given opportunities to ask questions before and after the interview. No limitations were observed during the remote interviews; however some individuals, such as those who are digitally excluded, may have been unable to participate.

While the sample size for this qualitative study was reasonable, it is beyond the scope of this research to offer broader recommendations for public health.

Finally, we acknowledge a limitation in that as this study took place during a pandemic and specifically during the third national lockdown during December 2020 and March 2021, it was not possible to conduct PPI activities. Although to some extent this was mitigated by engagement with the public health team at the local authority, such a lack of PPI may have impacted on various aspects of the study’s design and implementation research (eg, recruitment, topic guide, analysis, interpretation, etc). It is important that future post-pandemic studies, including those on the impact of covid on coastal communities, should engage in meaningful PPI strategies.

## Conclusion/implications for future research

Residents of East Sussex have been impacted by COVID-19 widely, differentially and at different time points during three lockdowns in the UK since March 2020. Mental health issues cross-cut many of the findings including withdrawal, feelings of social isolation, depression, and anxiety. The study shows that in many cases participants have been resilient in their response to the impacts of the pandemic using a range of positive behaviours and activities. As the long-term psychosocial impacts of the COVID-19 pandemic continue to unfold, further exploration of the post-pandemic impacts on coastal communities, including people with intersecting vulnerabilities, may help to refresh but also inform strategic priorities in a way that addresses both the negative aspects of the pandemic and builds on the positives, to promote greater health equity. Given the wide impact of the pandemic, local government is well placed to monitor and address this through various programmes. This includes working in partnership with healthcare providers and a range of partners within education, housing, skills and employment to address the wider determinants of health. This could inform a national strategy to improve the health and well-being of coastal communities.^8^,^10^

## Supplementary material

10.1136/bmjopen-2025-102031 online supplemental file 1

10.1136/bmjopen-2025-102031 online supplemental file 2

## Data Availability

No data are available.
