# Supplementary material for: Investigating the psychosocial impact of COVID-19 on coastal communities in East Sussex, UK: a qualitative analysis
Source: BMJ Open. 2025 Oct 23;15(10):e102031. doi: 10.1136/bmjopen-2025-102031 (PMC12557726; doi:10.1136/bmjopen-2025-102031)
Supplement: online supplemental file 1 [file bmjopen-15-10-s001.docx]

**Topic Guide**

**INTERVIEW SCHEDULE – IMPACT OF COVID-19 ON LOCAL COMMUNITIES WITHIN EAST SUSSEX**

*Note for interviewer: this is a generic interview schedule covering a range of impacts of the COVID-19 pandemic for a range of different participants. Therefore, some of these categories may not be applicable to the participant. This will need to be considered prior (and during) the interview.*

| Interviewer |  |
| --- | --- |
| Interviewee ID |  |
| Date |  |
| Location |  |

□ Introductory statement.

□ Received, read, understood PIS – Questions?

□ Consent form?

□ Recorder on?

**Introductory Statement (to be read only after recorder started)**

My name is (name of researcher) from the University of Brighton. I am calling you because you kindly agreed to take part in an interview about your experiences of the COVID-19 pandemic [briefly explain the project]. Are you still happy to do this?

We would like to explore your experiences since the COVID-19 outbreak, your beliefs about COVID-19, challenges you have faced since the COVID-19 outbreak, what has supported you since the COVID-19 outbreak and perhaps some of the positive things you may have done during the pandemic. The interview should last a maximum of 40 minutes. Everything you say will be confidential (unless you disclose information that could lead to harm for yourself or others) to the research team and will not be directly attributed to you. There are no right or wrong answers, we are just interested in hearing about your experiences.

**1) GENERAL PERCEPTIONS ABOUT COVID-19**

- Can you remember when you first heard about COVID-19?

*Probe. Initial thoughts, thoughts/feelings when first cases of COVID-19 were reported in the UK?*

- How do you feel about COVID-19 now?

*Probe. Has this changed from the start of the outbreak?*

- Are there things about COVID-19 that you would like to know more about?

*Probe. causes, transmission, signs, symptoms, safety measures, support, furlough etc*

- What safety measures have you taken for yourself (and your family) during COVID-19?

*Probe. Wearing a mask, social distancing, avoiding busy places, isolating/quarantining*

- Do you think you are at risk for COVID-19 infection?

*Probe. For any special considerations (age, occupation, pre-existing health conditions)*

- Have you been tested for COVID-19/diagnosed with COVID-19?

**2) EXPERIENCES OF THE COVID-19 OUTBREAK**

- What are your main concerns about the COVID-19 situation currently?

*Probe. Health of self/others, work, finances, education. How this has changed over the course of the pandemic/lockdowns*?

- Overall, how do you feel you are coping with the COVID-19 situation currently?

*Probe. If anything in particular they are having difficulty coping with. How has this changed over the course of the pandemic/lockdowns?*

- What are you most looking forward to doing when the COVID-19 restrictions and social distancing come to an end?

*Probe. Working on site, visiting family, holidays*

**3) IMPACTS OF THE COVID-19 PANDEMIC**

**General**

- Please can you tell me broadly about the impact of the COVID-19 pandemic on your life?

*Probe. How this has changed over the course of the pandemic/lockdowns?*

- What has been the greatest impact of COVID-19 restrictions and social distancing been on your life?

**Specific impacts** *[depending on the interviewee’s circumstances some of these impacts may not be explored]:*

***Impact on family life***

- Has the pandemic impacted your relationships with friends/family in your household?
- If yes, how have these relationships been impacted?

*Probe. Biggest impact, positive and negative impacts, unexpected impacts*

***Impact on friendships and relationships***

- Has the pandemic impacted your closest relationships?
- If yes, how have these relationships been impacted?

*Probe. Biggest impact, positive and negative impacts, unexpected impacts*

*(if applicable), How has shielding affected keeping in touch? Have you been able to keep in touch using technology like Zoom etc?*

***Impact on education***

- Has your (or someone in your household) education been impacted by COVID-19?

*Probe. Explore in what ways, perception of impacts (negative and positive)*

- How has COVID-19 changed the way you use the online learning environment and online resources?

***Impact on employment***

- Have you (someone in your household) lost your job/been furloughed since the COVID-19 pandemic?

*If yes, explore how long they have been unemployed/furloughed. Explore if full/part-time furlough, explore impacts of losing job/being furloughed*

*If no, ask if they are concerned about losing their job*

- Have your working practices been impacted by the COVID-19 situation?

*Probe. Keyworker, working from home full-time/part-time, Office worker at home, working online*

***Impact on finances***

- Have you/your household experienced a reduction in income due to COVID-19 (either due to you/someone in your household being furloughed, not able to work enough)?
- If yes, what is the scale of this reduction? What impact has this had on you/your household?

*Probe. Paying mortgage/rent/bills, buying essentials (food)*

If no, have the COVID-19 impacted on your finances in another way?

*Probe. Spending less money (going out less, no/fewer holidays)*

***Impact on social and leisure activities***

- Has the pandemic impacted on your social and leisure activities?
- If yes, which activities? Why have these been impacted? How have they been impacted?

*Probe. Biggest impact, positive and negative impacts, unexpected impacts*

- If no, why do you think this is?

***Impact on health behaviours***

- Do you think the pandemic has impacted on daily health behaviours?

*Probe. Smoking, alcohol, food (diet/nutrition), taking exercise*

- If yes, which behaviours? How have they been impacted?

*Probe. Biggest impact, positive and negative impacts, unexpected impacts*

- If no, why do you think this is?

***Impacts on housing***

a) Impacts of the COVID-19 pandemic on individuals living in temporary accommodation as a result of the “everybody in” initiative:

- Can you tell me about your experiences of being placed in temporary accommodation?

*Probe. What was happening before and what led up to it? What was that like?* *Explore knowledge of “everybody in” initiative; experiences of the services who provided the housing.*

- Can you tell me about your current housing situation?

*Probes. What kind of accommodation is it, e.g. hostel, council, housing association, B and B. What is it like living in temporary accommodation? Explore if they have been in temporary accommodation throughout the pandemic/lockdowns. Explore how long they think they will be staying here.*

- Please can you tell me about how moving to temporary accommodation has impacted you?

*Probe. Psychological, social, financial, positive, negative impacts, unexpected impacts; biggest impacts;*

- Do you have any concerns about your housing situation in the future?

*Probe. Explore what these are, explore biggest concern.*

b) Impacts of the COVID-19 pandemic on individuals living in temporary accommodation (for those who are in temporary accommodation for reasons other than the *“everybody in”* initiative):

- Could you tell me about your current housing situation?

*Probe. What kind of accommodation is it, e.g. hostel, council, housing association, B and B. What it is like living in temporary accommodation? Explore if they have been in temporary accommodation throughout the pandemic/lockdowns. Explore how long they think they will be staying here.*

- Has COVID-19 impacted your housing situation?

*Probe. security (e.g. feel able to stay longer)*

If yes, *explore impacts (positive, negative impacts, unexpected impacts), biggest impact*

- Do you have any concerns about your housing situation in the future?

*Probe. Explore what these are, explore biggest concern.*

***Impact on mental health and wellbeing***

- Have you had any concerns about your emotional wellbeing during the outbreak?
- Probe. *If yes, explore what these concerns are. And what do you think are the reasons for this, thinking about some of the previous things we’ve talked about?*
- If yes, have you sought help about these concerns?
- *Probe if yes. Type of help, experiences of seeking help (e.g. facilitators and challenges to seeking help)*
- *Probe if no. explore why help has not been sought*
- Has the COVID-19 restrictions and social distancing led to any benefits or positive outcomes for you or members of your family?
- *Probe. More time with family, more time for interests/hobbies*

***Other impacts (if not covered in previous sections)***

- Has the COVID-19 situation impacted on how you travel?

*Probe. Use of public transport, cycling, walking, driving*

**4) ACCESS TO HEALTHCARE OR PROFESSIONAL SUPPORT**

- Have you accessed/tried to access any healthcare services or professional support services since the outbreak?

*Probe. If yes, explore which and experiences of these services. Have you wanted/needed to access healthcare services but for whatever reason have not?*

- Have you experienced any changes or disruption to these healthcare/professional support services due to the COVID-19 pandemic?

*Probe. cancelled outpatients or appointments, cancelled operations, access to GP, collection of medication at pharmacy, views on telemedicine.*

- How confident do you currently feel about accessing healthcare/professional support services for any advice/support/treatment that are not COVID-19 related?

**5) AVAILABLE SUPPORT**

- In an ideal world, what support would you have/be able to access to help you (and your family) cope with the COVID-19 situation?
- What sort of help or support actually is accessible to you (and your family) to cope with the COVID-19 situation?
- Have other people had things that would have helped you?

**6) CONCLUSIONS**

- Is there anything else that you would like to tell me about regarding the impact on COVID-19 on you and your household?

**End**
