# Supplementary material for: Investigating the psychosocial impact of COVID-19 on coastal communities in East Sussex, UK: a qualitative analysis
Source: BMJ Open. 2025 Oct 23;15(10):e102031. doi: 10.1136/bmjopen-2025-102031 (PMC12557726; doi:10.1136/bmjopen-2025-102031)
Supplement: online supplemental file 2 [file bmjopen-15-10-s002.docx]

**Sample characteristics**

| **Mean**  **Age**  **(Range)** | **Gender** | **N (%)** | **Ethnicity** | **N**  **(%)** | **Sexual Orientation** | **N (%)** | **Location** | **N (%)** | **Relationship status** | **N (%)** | **Highest education** | **N (%)** | **Employment** | **N (%)** |
| --- | --- | --- | --- | --- | --- | --- | --- | --- | --- | --- | --- | --- | --- | --- |
| 45.3  (18-71) | Male | 15  (60) | White British | 19  (76) | Heterosexual | 19  (76) | Lewes District | 7  (28) | Married/Civil partnership | 9  (36) | GCSEs or equivalent | 1 (4) | Employed  FT | 7  (28) |
|  | Female | 10  (40) | White Other | 2  (8) | Bisexual | 3  (12) | Eastbourne District | 5  (20) | Living with partner | 1  (4) | A-Levels or equivalent | 9 (36) | Employed  PT | 7  (28) |
|  |  |  | Asian | 2  (8) | Lesbian | 1  (4) | Wealden District | 6  (24 | In a relationship | 1  (4) | Undergrad. Degree | 10 (40) | Self-employed | 4  (16) |
|  |  |  | Asian British | 1  (4) | Other | 1  (4) | Hastings Borough | 4  (16 | Single | 11  (44) | Postgrad. Degree | 5 (20) | Unemployed | 7  (28) |
|  |  |  | White & Black African | 1  (4) | Prefer not to say | 1  (4) | Rother District | 3  (12) | Separated/  divorced | 3  (12) |  |  |  |  |
| **Totals N**  **(%)** | | **25**  **(100)** |  | **25**  **(100)** |  | **25**  **(100)** |  | **25**  **(100)** |  | **25**  **(100)** |  | **25**  **(100)** |  | **25**  **(100)** |
