# Supplementary material for: Investigating the psychosocial impact of COVID-19 on coastal communities in East Sussex, UK: a qualitative analysis
Source: BMJ Open. 2025 Oct 23;15(10):e102031. doi: 10.1136/bmjopen-2025-102031 (PMC12557726; doi:10.1136/bmjopen-2025-102031)
Supplement: Supplementary file 3 [file bmjopen-2025-102031.reviewer_comments.pdf]

PEER REVIEW HISTORY

BMJ Open publishes all reviews undertaken for accepted manuscripts. Reviewers are asked to complete a checklist review form and are provided with free text boxes to elaborate on their assessment. These free text comments are reproduced below.

ARTICLE DETAILS

Title (Provisional)

Investigating the psychosocial impact of COVID-19 on coastal communities in East Sussex, UK: a qualitative analysis.

Authors

Sherriff, Nigel; Sawyer, Alexandra; Zeeman, Laetitia; Coleman, Lester; Kennedy, Sarah; Thomas, Jane; Bernhaut, Joanne; Salami-Oru, Teresa; Gale, Darrell

---

VERSION 1 - REVIEW

Reviewer 1

Name tanveer, sandila

Affiliation University of Otago Christchurch, Department of Psychological Medicine

Date 16-May-2025

COI None

This qualitative study offers valuable insights into how COVID-19 impacted the mental health, social lives, and wellbeing of diverse adults in coastal East Sussex communities. Through purposive sampling and in-depth thematic analysis of 25 interviews, it captures nuanced narratives during the UK's third lockdown. The study's strength lies in its contextual richness, diversity of participants, and relevance to public health planning for vulnerable coastal populations.

There are a few limitations which needs to be addressed.

1. Introduction: While the acute phase of the COVID-19 pandemic has passed, its long-term psychosocial impacts continue to unfold, especially among vulnerable communities. The authors are encouraged to contextualise their findings within this evolving landscape and reflect on how the lingering effects of the pandemic shape the relevance of their results today and in future pandemics.

The introduction, while informative, lacks a clearly articulated rationale and fails to identify a specific research gap. A concise summary of prior qualitative research on COVID-19—

explaining its importance and how the present study builds upon or differs from existing work—is necessary to establish the study's contribution.

2. Methodology

• Interview Prompt: The development process of the interview prompt remains unclear, raising questions about how the questions were shaped and whether they were informed by previous literature or theoretical models. Relevant citations in the introduction that align with the interview guide and anticipated themes would strengthen the theoretical grounding of the study.

• Sample Size and Saturation: The sample (n=25) is reasonable for qualitative research, but the manuscript does not mention if data saturation was achieved. Add a statement about whether thematic saturation was reached or considered sufficient.

• Sampling Limitations and Exclusion of Highly Vulnerable Groups: While the study included participants from a range of demographic backgrounds, it explicitly notes the exclusion of care home residents, asylum seekers, refugees, and Gypsy, Roma and Traveller communities (p. 4, 18). These are among the most affected and underrepresented populations. Acknowledge the exclusion of these groups as a key limitation. Also, recommend that future studies prioritize including such populations to capture a fuller range of psychosocial impacts.

• Language Restriction and Digital Divide: Only English-speaking participants were included due to time/resource constraints, potentially excluding non-English-speaking minorities, recent immigrants, and older adults with language or literacy barriers. In addition, online and telephone interviews may have excluded participants with poor digital access or skills, limiting representativeness. Discuss the digital exclusion as a limitation and recommend multilingual, mixed-method approaches for future work.

• Online Interview Limitation (p. 9): While safety justified remote interviews, the impact on rapport and depth of responses could be acknowledged more critically. Expand on how rapport was maintained and any observed limitations of remote interviews (especially for marginalized groups).

• Participant Recruitment Bias: Reliance on networks, social media, and professional contacts may introduce selection bias, favoring more connected or articulate individuals. Discuss the limitation of potential underrepresentation of those with no digital access or less connected to services.

• Limited Generalisability: The findings are not generalisable beyond the study context due to its small, geographically bounded sample (n=25) in East Sussex and a qualitative design. Emphasise that while in-depth, the insights are exploratory and not generalisable to all UK coastal communities or urban populations.

• Limited Patient and Public Involvement (PPI): The manuscript acknowledges that PPI was limited due to pandemic conditions (p. 4), yet this affects the study's relevance and

community accountability. Reflect on how this shaped the design or interpretations, and recommend meaningful PPI strategies for post-pandemic research.

3. Thematic Presentation and Depth

• Limited Analytical Depth: While the study presents rich descriptive data, it lacks a theoretical lens (e.g., resilience theory, ecological systems, or vulnerability frameworks) that could deepen the interpretation of findings and support transferability. Introduce brief theoretical insights in the discussion to enhance interpretive richness.

• Theme Development Transparency: It is unclear how themes were derived from coded data. There's a brief mention of two researchers summarizing data and a third deriving themes (p. 9), but no audit trail or coding structure is shared. Add a supplementary file or brief table outlining main themes, subthemes, and sample codes.

• Ambiguity Around Theme Saturation and Coding Rigor: The manuscript does not explicitly state whether thematic saturation was reached or how inter-coder reliability was ensured. Clarify whether saturation was a goal or achieved. If not assessed, note this as a methodological limitation. Recommend use of coding audits or intercoder consensus in future studies.

• Inconsistent Use of Subheadings and Thematic Categories: The results and discussion sections use overlapping but not always aligned thematic categories, making it harder for readers to trace findings across the manuscript. Improve coherence between thematic structure in the results and discussion sections, or note this as a presentation limitation.

• Missing Discussion of Intersectionality: While the sample includes gender, ethnicity, and employment diversity, the intersections of identity (e.g., how being LGBTI and unemployed, or shielding and elderly) are not explicitly analysed. Recommend that future qualitative research incorporate an intersectional analysis to better understand layered vulnerabilities.

4. Discussion

• Rather than offering a critical synthesis of findings in light of existing literature, the discussion often reads as an extension of the introduction. A clearer analytical tone is needed, highlighting what was found, how it relates to existing research, and why it matters in the current post-pandemic recovery context.

• The discussion section does not include a demarcated limitations subsection, nor does it offer concrete policy recommendations or future research directions.

NOTE: The abstract and conclusion sections should be updated reflecting these changes.

This paper offers a valuable contribution to qualitative pandemic research by highlighting the complex, multifaceted psychosocial experiences of coastal residents in the UK. The methodology is appropriate, the data are rich, and the findings relevant. However, several aspects—particularly the analytical depth, transparency in coding, reflexivity, and theoretical framing—should be strengthened to elevate the manuscript's scholarly impact.

**Reviewer**	**2**
**Name**	**Alchieri, João**
**Affiliation**	**Federal University of Rio Grande do Norte, Department of Psychology, Graduate Program in Science**
**Date**	**17-Jul-2025**
**COI**	**None**

The manuscript, Investigating the psychosocial impact of COVID-19 on coastal communities in East Sussex, UK: a qualitative analysis presents the study conducted in a devolved community in England during the COVID-19 pandemic. This is a qualitative, descriptive, cross-sectional study whose objective was to verify, from the perception of people in this coastal location, the aspects concerning the pandemic, basically based on psychosocial factors. The originality of the proposal on the psychosocial aspects as well as the sample composition in 2 levels using snowball techniques is verified. The authors present a manuscript in a scientific, objective language supported by diversified and current theoretical references, enabling the reader to understand different scopes.

The methodological aspects present details and descriptions capable of being reproduced in other environments and/or cultures. An instrument used is presented, enabling the reader to understand the time needed for investigation, both in data collection and in its analysis. The methodology is based on technical precepts recommended by international literature. Unfortunately, sensitive information does not allow the review of the data from the original interviews, but it is a fact understandable to the reviewer.

The analysis of the results is presented characterizing the sample and detailing sociodemographic and psychosocial information of the participants, so that the reader can understand the origin of the information. The authors describe levels of analysis, how I understood early memories of COVID-19; reduction of the risk of transmission; concerns about transmission to others; early impacts of COVID-19; specific impacts of COVID-19, family, friends and leisure; health-related behavior; mental health; health and social assistance appointments and jobs, characterizing the orientation in the process of participants' perception of the pandemic. It is an analysis strategy that facilitates the reader's understanding, although it has been somewhat superficial with the presentation of vignettes by some participants.

The discussion of the results is based on an extensive updated review of the literature on the pandemic and related aspects, enabling the reader to follow and understand the authors' understanding, being an important contribution of the manuscript. The varied themes in relation to the different characteristics of the psychosocial aspects present a more realistic panorama of the pandemic process for this portion of the population represented in the sample group.

The conclusions summarize the manuscript, presenting sometimes different indications of the perception of the effects of COVID-19, such as positive characterizations of the lockdown period. The authors demonstrate a critical understanding of the limitations of their study and enable options for rectifications of these.

The reviewer considers the theme of understanding the characteristics experienced in the last pandemic to be important, as possibilities of understanding for interventions in future episodes.

VERSION 1 - AUTHOR RESPONSE

Reviewer: 1
Dr. sandila tanveer, University of Otago Christchurch
Reviewer 1 comment
This qualitative study offers valuable insights into how COVID-19 impacted the mental health, social lives, and wellbeing of diverse adults in coastal East Sussex communities. Through purposive sampling and in-depth thematic analysis of 25 interviews, it captures nuanced narratives during the UK's third lockdown. The study's strength lies in its contextual richness, diversity of participants, and relevance to public health planning for vulnerable coastal populations.
Author response: Thank you for reviewing our paper, it is appreciated.

Reviewer 1 comment
There are a few limitations which needs to be addressed.
1. Introduction: While the acute phase of the COVID-19 pandemic has passed, its long-term psychosocial impacts continue to unfold, especially among vulnerable communities. The authors are encouraged to contextualise their findings within this evolving landscape and reflect on how the lingering effects of the pandemic shape the relevance of their results today and in future pandemics. The introduction, while informative, lacks a clearly articulated rationale and fails to identify a specific research gap. A concise summary of prior qualitative research on COVID-19—explaining its importance and how the present study builds upon or differs from existing work—is necessary to establish the study's contribution.
Author response. Done. Thank you for the comment. We have strengthened this aspect, by concisely articulating better the rationale that is in the manuscript.

Reviewer 1 comment
2. Methodology
• Interview Prompt: The development process of the interview prompt remains unclear, raising questions about how the questions were shaped and whether they were informed by previous literature or theoretical models. Relevant citations in the introduction that align with the interview guide and anticipated themes would strengthen the theoretical grounding of the study.
Author response: Done. The interview guide was developed collaboratively by the research team in partnership with the public health team at East Sussex County Council. This was particularly important to ensure that individual, community, and wider public health impacts were captured reflecting the social and wider social determinants of health (e.g. education, employment, housing). The guide was reviewed and refined iteratively after each interview. Designing the schedule in this way, meant it was intentionally broad to capture a wide range of

impacts of the COVID-19 pandemic across diverse participants. We have added some clarification about this in the manuscript, thanks for highlighting

Reviewer 1 comment
• Sample Size and Saturation: The sample (n=25) is reasonable for qualitative research, but the manuscript does not mention if data saturation was achieved. Add a statement about whether thematic saturation was reached or considered sufficient.
Author response: Done. We have now added a statement about this

Reviewer 1 comment
• Sampling Limitations and Exclusion of Highly Vulnerable Groups: While the study included participants from a range of demographic backgrounds, it explicitly notes the exclusion of care home residents, asylum seekers, refugees, and Gypsy, Roma and Traveller communities (p. 4, 18). These are among the most affected and underrepresented populations. Acknowledge the exclusion of these groups as a key limitation. Also, recommend that future studies prioritize including such populations to capture a fuller range of psychosocial impacts.
Author response: Done. We do already comment on this exclusion issue in the discussion, and have noted the priority of capturing the long-term impacts of COVID-19 across these vulnerable communities.

Reviewer 1 comment
• Language Restriction and Digital Divide: Only English-speaking participants were included due to time/resource constraints, potentially excluding non-English-speaking minorities, recent immigrants, and older adults with language or literacy barriers. In addition, online and telephone interviews may have excluded participants with poor digital access or skills, limiting representativeness. Discuss the digital exclusion as a limitation and recommend multilingual, mixed-method approaches for future work.
Author response: Done. We have addressed this both in the data analysis section as well as in the discussion.

Reviewer 1 comment
• Online Interview Limitation (p. 9): While safety justified remote interviews, the impact on rapport and depth of responses could be acknowledged more critically. Expand on how rapport was maintained and any observed limitations of remote interviews (especially for marginalized groups).
Author response: Done. We have added this into the limitations

Reviewer 1 comment
• Participant Recruitment Bias: Reliance on networks, social media, and professional contacts may introduce selection bias, favoring more connected or articulate individuals. Discuss the limitation of potential underrepresentation of those with no digital access or less connected to services.
Author response: Done. We have addressed as part of the previous comment.

Reviewer 1 comment
• Limited Generalisability: The findings are not generalisable beyond the study context due to its small, geographically bounded sample (n=25) in East Sussex and a qualitative design. Emphasise that while in-depth, the insights are exploratory and not generalisable to all UK coastal communities or urban populations.
Author response: Done. It's unusual to comment on generalisability in qualitative research, rather we have therefore noted in the limitations how although this study provides in-depth

insight into the experiences of a diverse sample of individuals impacted by the covid-19 pandemic, the findings may not be fully transferrable to coastal communities in other regions of the UK.

Reviewer 1 comment
• Limited Patient and Public Involvement (PPI): The manuscript acknowledges that PPI was limited due to pandemic conditions (p. 4), yet this affects the study's relevance and community accountability. Reflect on how this shaped the design or interpretations, and recommend meaningful PPI strategies for post-pandemic research.
Author response: Done. Thanks this is a fair point and useful comment. We have added some additional detail to the existing PPI statement in the methods and have noted this as an additional limitation in the discussion.

Reviewer 1 comment
3. Thematic Presentation and Depth
• Limited Analytical Depth: While the study presents rich descriptive data, it lacks a theoretical lens (e.g., resilience theory, ecological systems, or vulnerability frameworks) that could deepen the interpretation of findings and support transferability. Introduce brief theoretical insights in the discussion to enhance interpretive richness.
Author response: Thanks for the comment. We have not actioned this as it would in our view, mean going beyond what the research was intended or designed to do; to investigate qualitatively the psychosocial impact of COVID-19 on coastal communities in East Sussex. Our research was not intended to theorise or use such a proposed theoretical lens regarding impacts.

Reviewer 1 comment
• It is unclear how themes were derived from coded data. There's a brief mention of two researchers summarizing data and a third deriving themes (p. 9), but no audit trail or coding structure is shared. Add a supplementary file or brief table outlining main themes, subthemes, and sample codes.
Author response: Thanks for the comment. We have not actioned this comment as we simply did not use a framework approach or coding table in the way that is implied here.
Reviewer 1 comment
Ambiguity Around Theme Saturation and Coding Rigor: The manuscript does not explicitly state whether thematic saturation was reached or how inter-coder reliability was ensured. Clarify whether saturation was a goal or achieved. If not assessed, note this as a methodological limitation. Recommend use of coding audits or intercoder consensus in future studies.
Author response: Done. We have addressed this in response to an earlier comment but also added some additional details around saturation. We have also noted that we did not assess coding reliability or intercoder consensus, as these approaches are more aligned with positivist paradigms and are not always appropriate in qualitative research depending on the approach taken. We therefore do not agree that these are methodological limitations, more they were not appropriate within the analytic approach we took (Braun and Clarke, 2006).

Reviewer 1 comment
Inconsistent Use of Subheadings and Thematic Categories: The results and discussion sections use overlapping but not always aligned thematic categories, making it harder for readers to trace findings across the manuscript. Improve coherence between thematic structure in the results and discussion sections, or note this as a presentation limitation.
Author response: Done. Thank you for highlighting this. Subheadings used in the results are now better aligned to the discussion.

Reviewer 1 comment
• Missing Discussion of Intersectionality: While the sample includes gender, ethnicity, and employment diversity, the intersections of identity (e.g., how being LGBTI and unemployed, or shielding and elderly) are not explicitly analysed. Recommend that future qualitative research incorporate an intersectional analysis to better understand layered vulnerabilities.
Author response: Thank you for this important comment. We recruited a diverse sample of residents varying in socio-demographic characteristics as well as social and economic circumstances or situations. We have added some text around this and noted it as a limitation and identifies it as an area for future research.

Reviewer 1 comment
4. Discussion
• Rather than offering a critical synthesis of findings in light of existing literature, the discussion often reads as an extension of the introduction. A clearer analytical tone is needed, highlighting what was found, how it relates to existing research, and why it matters in the current post-pandemic recovery context.
Author response: Done. We have amended this section

Review 1 comment to the Author:
• The discussion section does not include a demarcated limitations subsection, nor does it offer concrete policy recommendations or future research directions.
Author response: Done. Strengths and limitations were already included in the manuscript, however, we have added a subtitle to make this clearer. Future research directions have now been added.
NOTE: The abstract and conclusion sections should be updated reflecting these changes.
This paper offers a valuable contribution to qualitative pandemic research by highlighting the complex, multifaceted psychosocial experiences of coastal residents in the UK. The methodology is appropriate, the data are rich, and the findings relevant. However, several aspects—particularly the analytical depth, transparency in coding, reflexivity, and theoretical framing—should be strengthened to elevate the manuscript's scholarly impact.
Author response: The relevant sections have been updated to reflect the suggested changes

Reviewer: 2
Prof. João Alchieri, Federal University of Rio Grande do Norte

Reviewer 2 comment:
The manuscript, Investigating the psychosocial impact of COVID-19 on coastal communities in East Sussex, UK: a qualitative analysis presents the study conducted in a devolved community in England during the COVID-19 pandemic. This is a qualitative, descriptive, cross-sectional study whose objective was to verify, from the perception of people in this coastal location, the aspects concerning the pandemic, basically based on psychosocial factors. The originality of the proposal on the psychosocial aspects as well as the sample composition in 2 levels using snowball techniques is verified. The authors present a manuscript in a scientific, objective language supported by diversified and current theoretical references, enabling the reader to understand different scopes.
Author response: Thank you for reviewing our paper, it is appreciated.

Reviewer 2 comment:
The methodological aspects present details and descriptions capable of being reproduced in other environments and/or cultures. An instrument used is presented, enabling the reader to

understand the time needed for investigation, both in data collection and in its analysis. The methodology is based on technical precepts recommended by international literature. Unfortunately, sensitive information does not allow the review of the data from the original interviews, but it is a fact understandable to the reviewer.
The analysis of the results is presented characterizing the sample and detailing sociodemographic and psychosocial information of the participants, so that the reader can understand the origin of the information. The authors describe levels of analysis, how I understood early memories of COVID-19; reduction of the risk of transmission; concerns about transmission to others; early impacts of COVID-19; specific impacts of COVID-19, family, friends and leisure; health-related behavior; mental health; health and social assistance appointments and jobs, characterizing the orientation in the process of participants' perception of the pandemic. It is an analysis strategy that facilitates the reader's understanding, although it has been somewhat superficial with the presentation of vignettes by some participants.
Author response: Thank you for the comment.

Reviewer 2 comment:
The discussion of the results is based on an extensive updated review of the literature on the pandemic and related aspects, enabling the reader to follow and understand the authors' understanding, being an important contribution of the manuscript. The varied themes in relation to the different characteristics of the psychosocial aspects present a more realistic panorama of the pandemic process for this portion of the population represented in the sample group. The conclusions summarize the manuscript, presenting sometimes different indications of the perception of the effects of COVID-19, such as positive characterizations of the lockdown period. The authors demonstrate a critical understanding of the limitations of their study and enable options for rectifications of these. The reviewer considers the theme of understanding the characteristics experienced in the last pandemic to be important, as possibilities of understanding for interventions in future episodes.
Author response: Thank you for the comment.

VERSION 2 - REVIEW

**Reviewer**	**1**
**Name**	**tanveer, sandila**
**Affiliation**	**University of Otago Christchurch, Department of Psychological Medicine**
**Date**	**01-Oct-2025**
**COI**	

I appreciate the authors' efforts in revising the manuscript and thoughtfully addressing the reviewers' comments. I wish them all the best in their future research endeavors.
